# Dipotassium 1,3,4-thiadiazole-2,5-bis(thiolate) as a new S-donor for direct synthesis of symmetrical disulfides

**DOI:** 10.1038/s41598-022-20642-5

**Published:** 2022-09-27

**Authors:** Mohammad Soleiman-Beigi, Mohammad Alikarami, Homa Kohzadi, Zahra Akbari

**Affiliations:** 1 Department of Chemistry, Faculty of Science, Ilam University, P.O. Box 69315516, Ilam, Iran; 2 Department of Chemistry, Ilam Branch, Islamic Azad University, Ilam, Iran

**Keywords:** Chemistry, Organic chemistry, Reaction mechanisms

## Abstract

In this research, a simple, efficient and novel protocol is eveloped for the direct synthesis of symmetrical disulfides using dipotassium 1,3,4-thiadiazole-2,5-bis(thiolate) as a new, low toxicity, inexpensive, stable solid and free of foul-smelling thiols for synthesize symmetric diaryl/dialkyl disulfides from aryl and alkyl halides in presence of MOF-199 and CuO nanoparticles. Significantly, using this method results in obtaining a variety of symmetrical disulfides in moderate to excellent yields (up to 98%).

## Introduction

Disulfides which are used in many organic procedures play a very important role as vulcanizing agents and linkages for controlled drug delivery. Furthermore, this compound has received significant attention because of being indispensable in many important synthetic chemistry, biochemistry and industrial applications^1–5^. Due to the importance of these compounds, various methods have been developed in order to prepare such compounds. The use of oxidative coupling of thiols with stoichiometric oxidation or catalytic oxidation has become a classical protocol for the synthesis of disulfides. In this sense, reagents such as 4,4′-azopyridine^6^, N-phenyltriazolinedione^7^, Fe(NO_3_)_3_, 9H_2_O/Fe(HSO_4_)_3_^8^, DDQ^9^, tributylammonium halochromates/silica gel^10^, Burgess reagent^11^, CAN^12^, bromate^13^ and N_2_O_4_/PVP^14^ have been used as stoichiometric oxidants. Although these methods are used for synthesis of disulfides, they have some specific disadvantages, i.e. long reaction time, difficult work-up, use of toxic or costly reagents, low yield of product due to over oxidation, etc. It is worth mentioning that some of the proposed methods have been devised with various sulfur-transfer agents, such as sulfonyl chlorides^15^, carbon disulfide^16^, 1,3-thiazolidinedione^17^, thiourea^18^ and sulfur^19^. In this regard, thiols have been used in most methods designed to synthesize organosulfurs. In addition, thiols are malodor, volatile, and toxic compounds. Thus, in order to overcome these problems, other sources and transporters of sulfur which are solid, stable and odorless have been introduced. Significantly, sulfur transporters play an effective role in the formation of carbon–sulfur (C-S) bonds.

**Figure 1:**
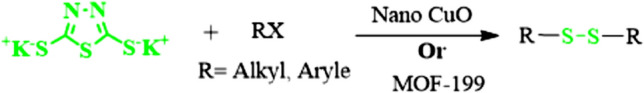
Synthesis of disulfides using MOF-199 and CuO nanoparticles.

Herein, in continuation of our researches, regarding the synthesis of organosulfur compounds^20–22^, a new method is reported for the synthesis of symmetric diaryl and dialkyl disulfides from aryl and alkyl halides using dipotassium 1,3,4-thiadiazole-2,5-bis(thiolate) as an excellent sulfur source in presence of CuO and MOF-199 nanoparticles (Fig. 1).

MOF-199 is a unique class of metal–organic frameworks which are known for their applications in various fields such as drug delivery, gas storage, semiconductors and catalysis^23,24^. Metal–organic frameworks (MOFs) due to their unique properties such as their exceptional porosity and high surface area have been wildly used as catalyst and, accordingly, have shown good potential in producing heterogeneous catalysis.

In this research we also used CuO nanoparticles as catalyst for the synthesis of diaryl and dialkyl disulfides. Nano-crystalline metal oxides with advantages such as high surface area and reactive morphologies can be exceptionally applied as catalysts for various organic transformations^25,26^.

After synthesizing MOF-199 and CuO nanoparticles, FT-IR, SEM, EDX and XRD analyses were used in order to characterize these nanoparticles.

## Results and discussion

**Figure 2:**
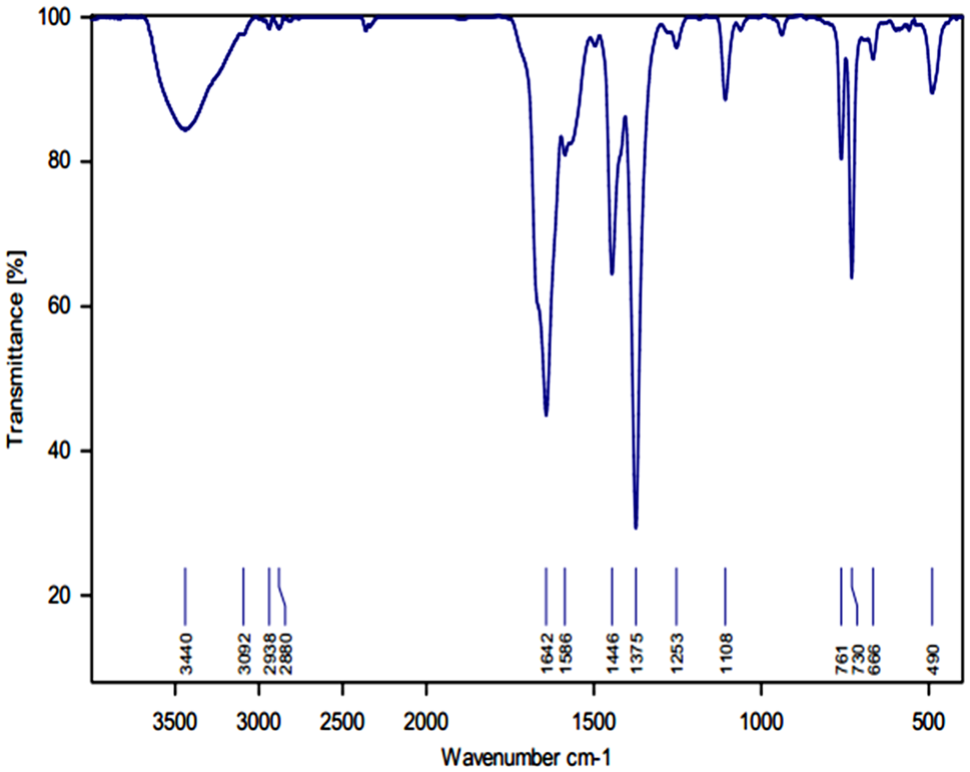
FT-IR spectrum of MOF-199.

**Figure 3:**
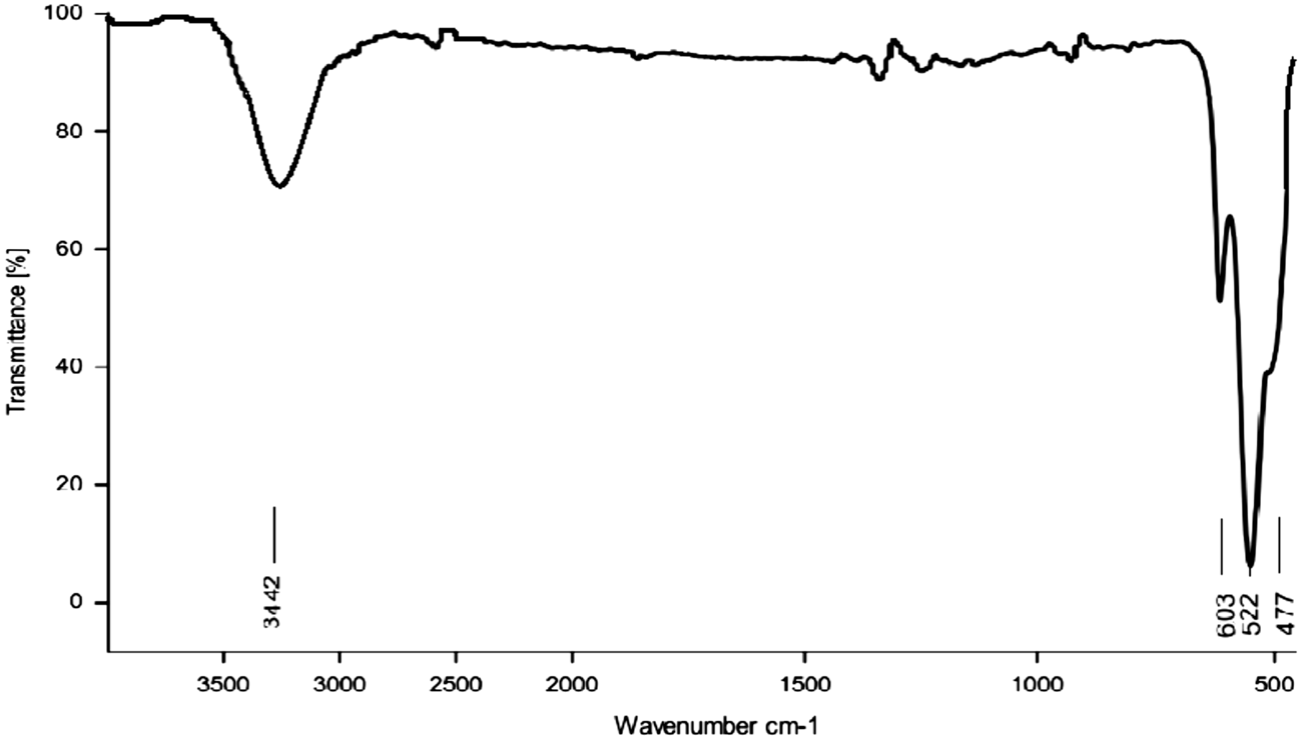
FT-IR spectrum of CuO nanoparticles.

The FT-IR spectrum of MOF-199 indicated three peaks at 3440 cm^−1^, 1642 cm^−1^ and 1446 cm^−1^ which belong to H_2_O, EtOH molecules in the cavities of the matter, carbonyl group of the benzene tricarboxylic acid and the double bond of the benzene ring, respectively (Fig. 2). Regarding the FT-IR of Cuo nanoparticles, the stretching vibrations at 477 cm^−1^, 522 cm^−1^ and 603 cm^−1^ can be related to the Cu–O band (Fig. 3).

**Figure 4:**
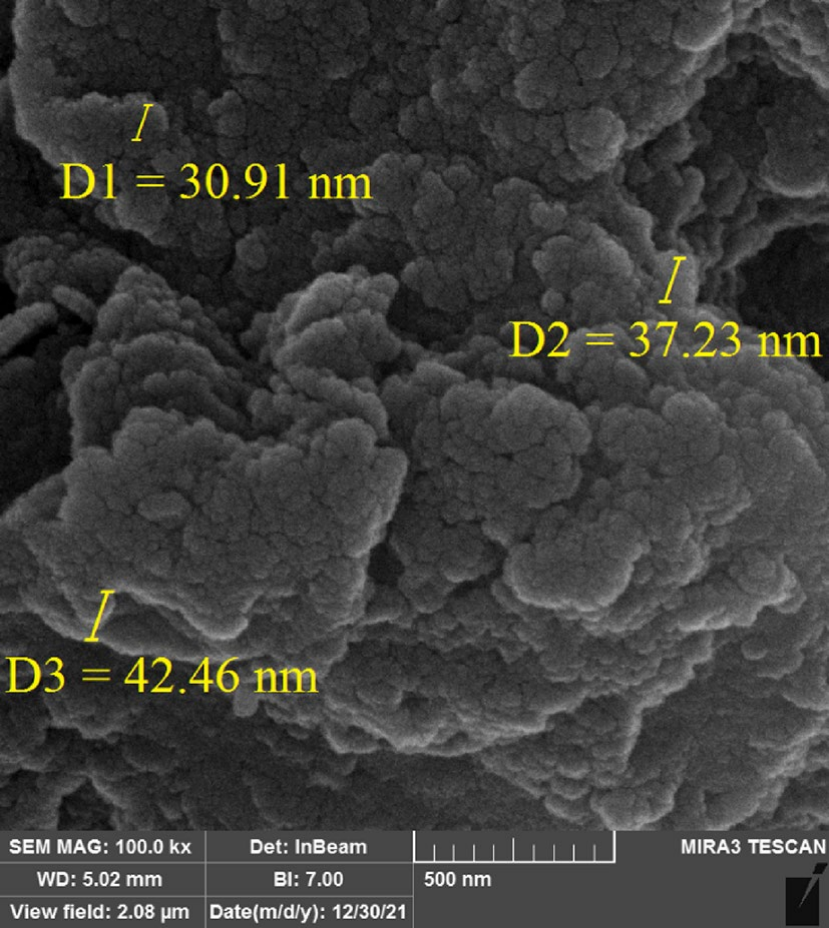
SEM image of MOF-199.

**Figure 5:**
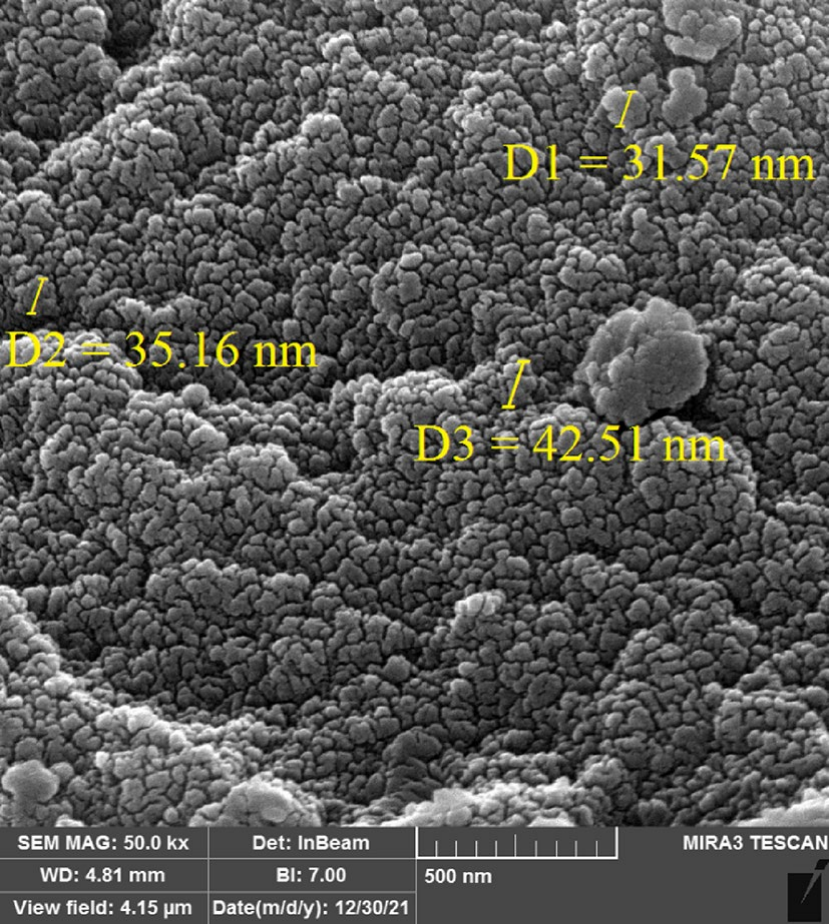
SEM image of CuO nanoparticles.

The morphology and size of MOF-199 and CuO nanoparticles are determined, using scanning electron microscopy technique. Moreover, SEM images show that the particle size of MOF-199 and CuO NPs are 30–40 nm (Figs. 4, 5).

**Figure 6:**
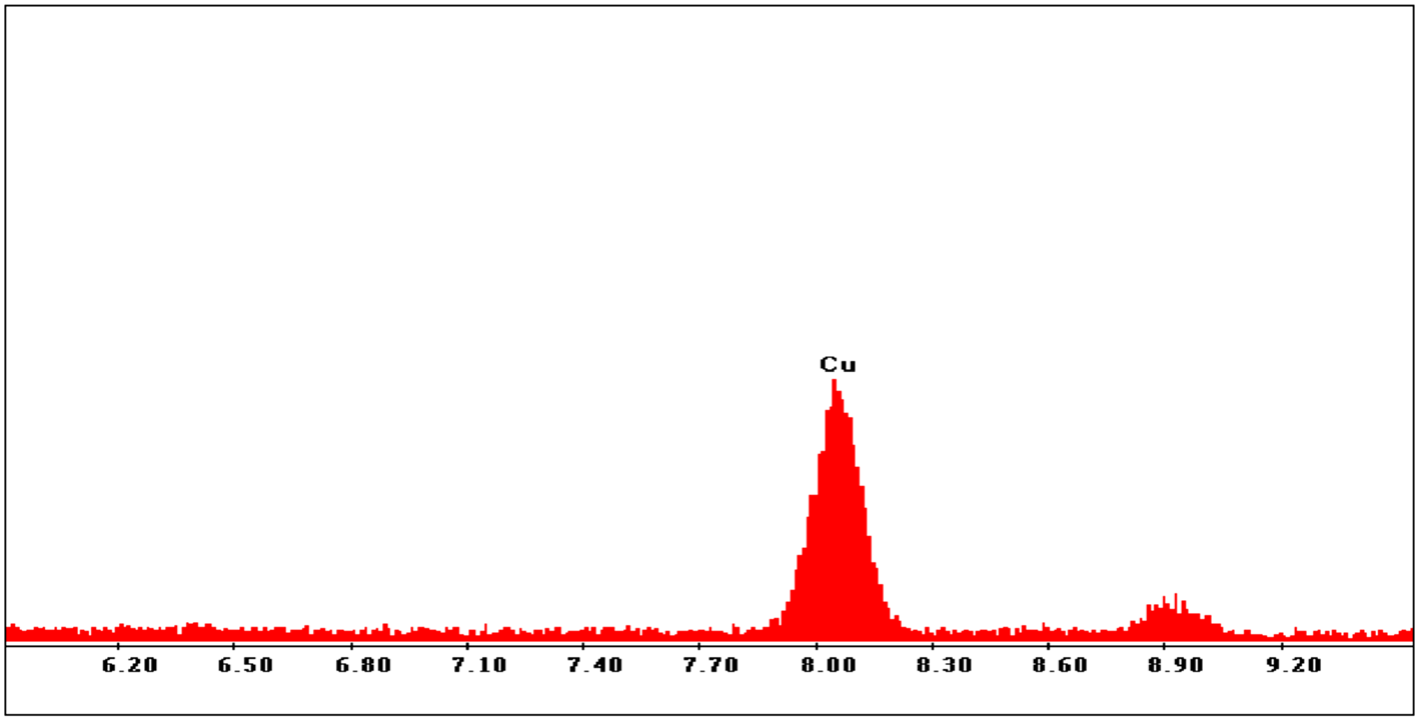
EDX spectrum of MOF-199.

**Figure 7:**
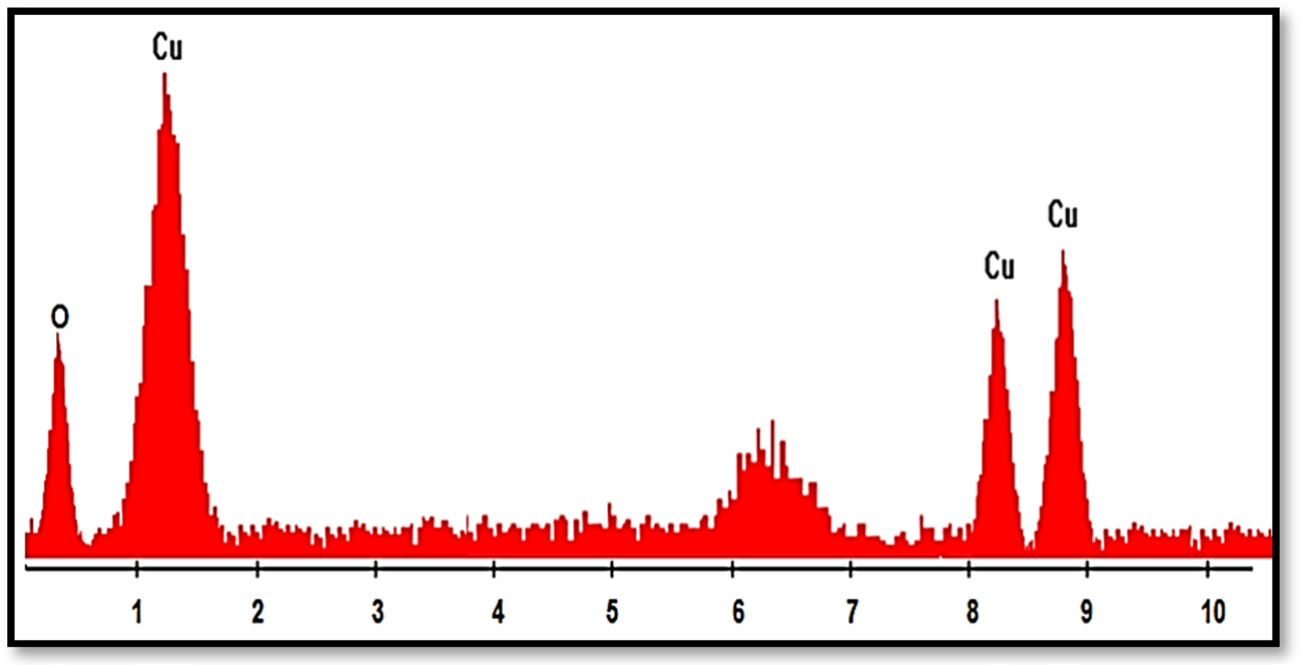
EDX spectrum of CuO nanoparticles.

One of the best approaches to determine of elements present in nanoparticles and the purity of nanoparticles is energy-dispersive X-ray spectroscopy (EDS). The EDX spectra of the MOF-199 and CuO NPs approve the presence of Cu and O elements in the structure of the catalysts and also confirm the fact that the nanoparticles have been successfully synthesized (Figs. 6,7).

**Figure 8:**
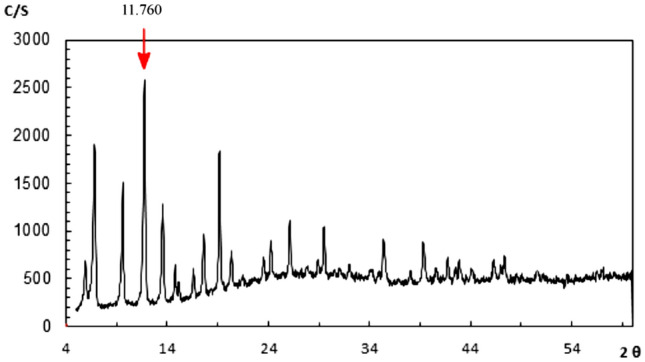
XRD of MOF-199.

**Figure 9:**
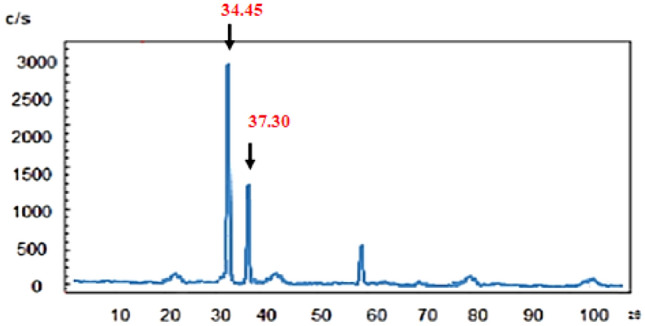
XRD of CuO nanoparticles.

X-ray diffraction (XRD) was used in order to investigate the structure of MOF-199 and CuO NPs. Regarding the XRD pattern of MOF-199, the biggest peak (222) is at 2θ = 11.76°. Considering the peaks observed in the region of 2θ = 10°–20° indicate the crystal structure of the MOF-199 metal–organic framework, which is consistent with the pattern presented in previous studies. (Fig. 8). Moreover, it is worth mentioning that 2θ = 34.45°, 37.30° for the CuO NPs confirms the structure of the catalyst (Fig. 9).

After the synthesis and characterization of MOF-199 and CuO NPs, the sulfur-transfer of 1,3,4-thiadiazole-2,5-bis(thiolate) was investigated in the synthesis of disulfides. In order to reach optimum conditions, iodobenzene and 1,3,4-thiadiazole-2,5-bis(thiolate) were used as a sulfur source in presence of different parameters including solvent, temperature and amounts of MOF-199 (method a) and CuO (method b). Based on the results of Table 1, the best results were obtained in dimethylformamide as a solvent using 4 mg MOF-199 and 5 mg CuO at 100 °C.

**Table 1:** Optimization of the reaction conditions^a^.


Entry	Sulfur Source (mmol)	Temp (°C)	Solvent (ml)		Cat (mg)		Time (h)		Yield (%)	
			a	b	a	b	a	b	a	b
1	1.5	100	PEG	DMF	4	5	7	8:30	85	82
2	2	100	PEG	DMF	4	5	4	5	98	98
3	2.5	100	PEG	DMF	4	5	5	6	90	87
4	2	100	PEG	DMF	3	4	6	9	85	87
5	2	100	PEG	DMF	6	7	5	8	87	94
6	2	100	H_2_O	H_2_O	4	5	24	24	45	30
7	2	100	PEG	PEG	4	5	4	8	58	65
8	2	100	PEG	DMF	4	5	6	5	87	98
9	2	reflux	EtOH	EtOH	4	5	10	9	80	85
10	2	r.t	PEG	DMF	4	5	4	5	–	–
11	2	45	PEG	DMF	4	5	4	5	66	60
12	2	75	PEG	DMF	4	5	4	5	70	65
13	2	120	PEG	DMF	4	5	4	5	90	87

a: Reaction conditions: iodobenzene (1 mmol), 1,3,4-thiadiazole-2,5-bis(thiolate), Temp (°C), 2 ml solvent, MOF-199.

b: Reaction conditions: iodobenzene (1 mmol), 1,3,4-thiadiazole-2,5-bis(thiolate), Temp (°C), 2 ml solvent, CuO NPs.

After obtaining the optimum conditions, a large number of symmetrical diaryl (alkyl) disulfides (1a–1l) was synthesized using 1,3,4-thiadiazole-2,5-bis(thiolate) in presence of MOF-199 and CuO NPs, the results of which are shown in Table 2. As can be seen, aryl iodides were more reactive than aryl bromides. Also based on results in Table 2, both catalysts have a good to excellent yields, but MOF-199 has more activity and efficiency for the synthesis of diaryl and dialkyl disulfides. In most cases, the yield of the reactions was higher with shorter reaction times in the presence of MOF-199.

**Table 2:** Synthesis of disulfides^a^.


Entry	Aryl/Alkyl halide	Time (h)		Yield (%)		m.p (°C). Ref
		a	b	a	b	
1	Iodobenzene (1a)	4	5	98	98	57–60^16^
2	Bromobenzene (1a)	7	8:30	88	90	58–61^16^
3	Chlorobenzene (1a)	8	9	85	83	60–62^16^
4	1-Iodo-2-methoxybenzene (1b)	9:30	10	85	90	119–120^27^
5	1-Iodo-4-methoxybenzene (1c)	11	9	75	85	41–43^28^
6	1-Bromo-4-iodobenzene(1d)	8	8	80	75	93–96^29^
7	1-Bromo-4-nitrobenzene (1e)	6	7	90	85	173–175^27^
8	1-Iodonaphthalene (1f.)	6	7	92	90	93–94^30^
9	1-Bromonaphthalene (1f.)	8	8:30	87	85	92–94^30^
10	2-Iodothiophene (1g)	7	5	92	90	52–54^31^
11	2-Bromothiophene (1g)	8:30	8	75	80	53–55^31^
12	Benzyl chloride (1h)	2:30	2	90	87	69–71^32^
13	2-Phenylethyl bromide (1i)	1	1:30	95	92	Oil^32^
14	(3-bromopropyl) benzene (1j)	1	1	98	95	Oil
15	4-Iodoaniline (1k)	9	7	87	80	73–76^33^
16	1-Iodo-2-methylbenzene (1l)	3	6	98	88	38–40^34^

a: Reaction conditions: aryl or alkyl halide (1 mmol), 1,3,4-thiadiazole-2,5-bis(thiolate) (2 mmol), MOF-199 (4 mg) 100 °C, DMF.

b: Reaction conditions: aryl or alkyl halide (1 mmol), 1,3,4-thiadiazole-2,5-bis(thiolate) (2 mmol), CuO (5 mg), 100 °C, PEG.

**Figure 10:**
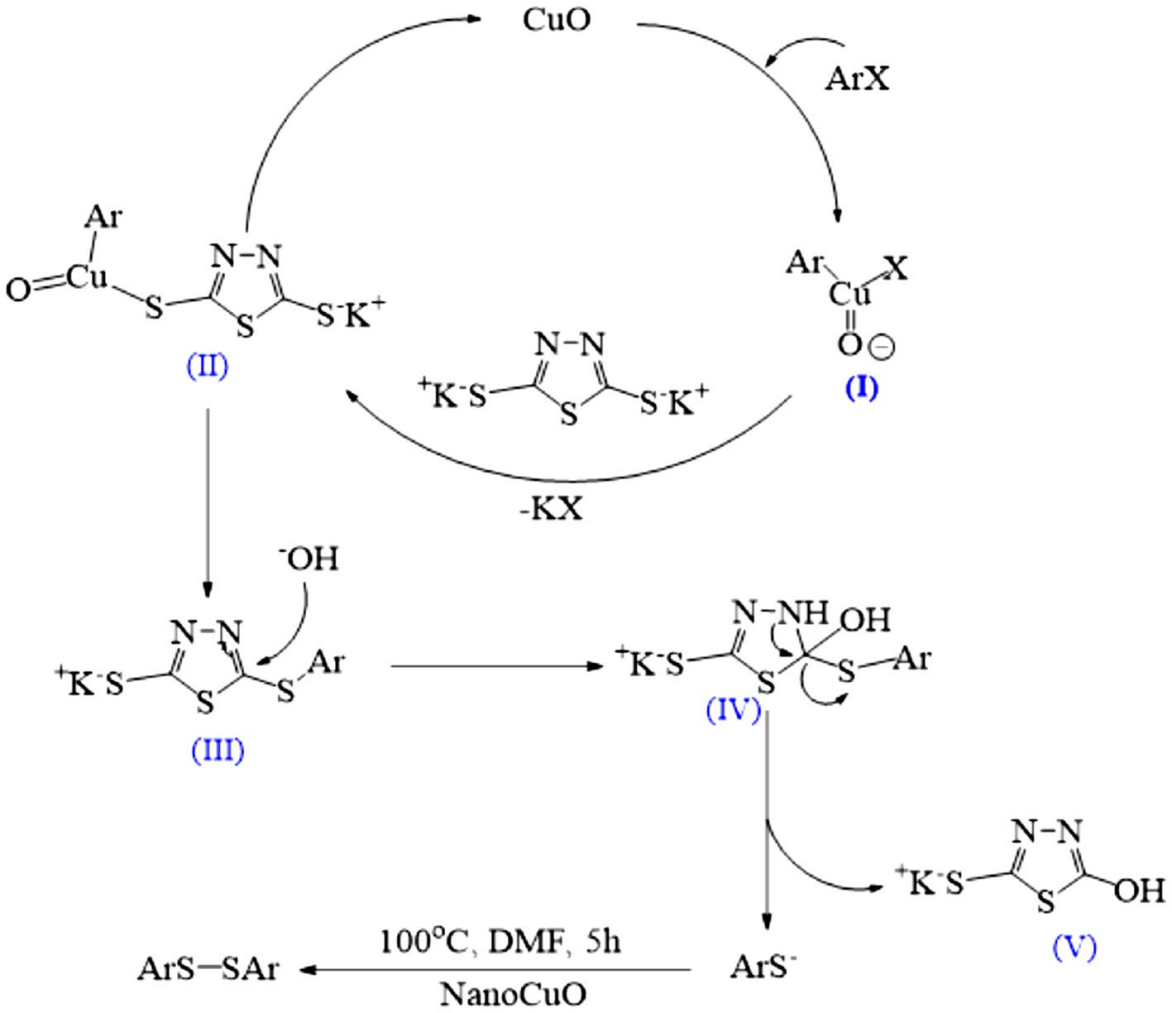
A possible mechanism for the synthesis of disulfides.

The possible mechanism for the synthesis of disulfides is shown in Fig. 10^21^. The mechanism of this reaction is similar to Ullmann's reaction. The first step is the oxidative addition of copper to the aryl halide, which the organocopper intermediate (I) is formed. Consequently, it reacts with 1,3,4-thiadiazole-2,5-bis(thiolate) and finally converts to intermediate (II). Afterwards, intermediate (III) is produced using CuO extraction. Subsequently, when hydroxide attacks intermediate (III), compound (IV) is produced. In the following, by exit of compound (V), the corresponding disulfide is synthesized under the reaction conditions.

## Experimental section

### Synthesis of MOF-199

Considering the synthesis of MOF-199, a mixture of benzene-1,3,5-tricarboxylic acid (2.38 mmol) and Cu(OAc)_2_.H_2_O (4.31 mmol) was added to the EtOH/H_2_O/DMF (1:1:1). Afterwards, Et_3_N (0.5 mmol) was added to the reaction mixture and, then, stirred for the 24 h at room temperature. After completion, the product was separated by filtration, washed with DMF for the several times and, finally, dried at 150 °C in oven.

### Synthesis of CuO nanoparticles

In order to synthesize CuO NPs, the solution of NaOH (100 ml, 0.1 M) was added dropwise to the 50 ml of Cu(OAc)_2_.2H_2_O (0.05 M) and, then, sonicated at 60 °C for the 45 min. Subsequently, 10 g PEG was solved in 10 ml distillated water and, then, it was added to the reaction mixture dropwise and sonicated for 1 h. Finally, the product was separated using centrifugation process and, then, dried at 45 °C in oven.

### Synthesis of dipotassium 1,3,4-thiadiazole-2,5-bis(thiolate)

In order to synthesize dipotassium 1,3,4-thiadiazole-2,5-bis(thiolate), a mixture of hydrazine hydrate (0.02 mmol), carbon disulfide (0.02 mmol) and pyridine (50 ml) was added to EtOH at room temperature for thirty minutes. Afterwards, the reaction mixture was stirred for the 5 h at 60 C^o^. After completion of the reaction, HCl (5 ml) was added to the reaction mixture and, then, the product (1,3,4-thiadiazole-2,5-dithiol) was separated by filtration, washed with EtOH for the several timed and dried at 80 °C in oven. In the next step, a mixture of 1,3,4-thiadiazole-2,5-dithiol (10 mmol) and KOH (20 mmol) was added to EtOH (15 ml) for 3 h at 40 °C. After completion of the reaction, the product was separated by filtration, washed with EtOH for the several times and dried at 50 °C in oven.

### General procedure for the synthesis of disulfides using MOF-199

A mixture of aryl or alkyl halide (1 mmol), 1,3,4-thiadiazole-2,5-bis(thiolate) (2 mmol) and MOF-199 (4 mg) in DMF (2 ml) was stirred at 100 °C. After completion of the reaction, the reaction mixture was cooled to room temperature and, then, the product was separated using H_2_O and EtOAc.

### General procedure for the synthesis of disulfides using CuO NPs

A mixture of aryl or alkyl halide (1 mmol), 1,3,4-thiadiazole-2,5-bis (thiolate) (2 mmol) and CuO (5 mg) in PEG (2 ml) was stirred at 100 °C. After completion of the reaction, the reaction mixture was cooled to room temperature and, then, the product was separated using H_2_O and EtOAc. The analysis of some compound contain 1H NMR and13C NMR spectrum are available in supplementary information.

*Diphenyl disulfide.* M.p. = 57–60 °C. ^1^H NMR (400 MHz, CDCl_3_): δ = 7.54–7.53 (m, 4H), 7.24–7.36 (m, 6H) ppm. ^13^C NMR (100 MHz, CDCl_3_): δ = 137.0, 129.1, 127.5, 127.2 ppm.

*Bis(4-methoxyphenyl) disulfide.* M.p. = 41–43 °C. ^1^H NMR (400 MHz, CDCl_3_): δ = 7.31 (d, *j* = 8.8 Hz, 4H), 6.87 (d, j = 8.8 Hz, 4H), 3.82 (s, 6H) ppm. ^13^C NMR (100 MHz, CDCl_3_): δ = 159.0, 132.8, 127.4, 114.7, 55.4 ppm.

*Bis(4-nitrophenyl) disulfide.* M.p. = 173–175 °C. ^1^H NMR (400 MHz, CDCl_3_): δ = 8.14 (d, j = 9.2 *H,* 4H), 6.62 (d, *j* = 9.2 *H,* 4H) ppm. ^13^C NMR (100 MHz, CDCl_3_): δ = 155.4, 138.1, 127.1, 113.8 ppm.

*Dibenzyl disulfide.* M.p. = 69–71 °C. ^1^H NMR (400 MHz, CDCl_3_): δ = 3.63 (s, 4H), 7.28–7.38 (m, 10H) ppm. ^13^C NMR (100 MHz, CDCl_3_): δ = 137.4, 129.5, 128.5, 127.5, 43.3 ppm.

## Conclusion

In conclusion, an effective method was reported for the synthesis of symmetric diaryl (dialkyl) disulfides from aryl and alkyl halides. The salient features of the present protocol include: being more economic, comprehensive and environmentally friendly than previous methods. Moreover, dipotassium 1,3,4-thiadiazole-2,5-bis(thiolate) was introduced as a new sulfur source for the synthesis of symmetric disulfides from aryl and alkyl halides in presence of MOF-199 and CuO nanoparticles. Dipotassium 1,3,4-thiadiazole-2,5-bis(thiolate) has great potential as a sulfur-transfer reagent and possesses some specific advantages such as: low toxicity, water-solubility, stability and being odorless. Therefore, this strategy provides a new method for the direct synthesis of symmetrical disulfides.

## Supplementary Information

Supplementary Information.

## Data Availability

All data generated or analyzed during this study are included in this published article [and its supplementary information files].

## References

[1] Dutta K, Das R, Medeiros J, Thayumanavan S (2021). Disulfide bridging strategies in viral and nonviral platforms for nucleic acid delivery. Biochemistry.

[2] Yokoyama K, Ogaya D, Utsumi H, Suzuki M, Kashiwagi T, Suzuki E, Taguchi S (2021). Effect of introducing a disulfide bridge on the thermostability of microbial transglutaminase from Streptomyces mobaraensis. Appl. Microbiol. Biotechnol..

[3] Grishin AM, Dolgova NV, Landreth S, Fisette O, Pickering IJ, George GN, Falzarano D, Cygler M (2022). Disulfide bonds play a critical role in the structure and function of the receptor-binding domain of the SARS-CoV-2 spike antigen. J. Mol. Biol..

[4] Wang F, Chen Y, Rao W, Ackermann L, Wang SY (2022). Efficient preparation of unsymmetrical disulfides by nickel-catalyzed reductive coupling strategy. Nat. Commun..

[5] Ding J, Feng A, Li X, Ding S, Liu L, Ren W (2021). Properties, preparation, and application of tungsten disulfide: A review. J. Phys. D Appl. Phys..

[6] Khalili D, Iranpoor N, Firouzabadi H (2015). 4, 4′-Azopyridine as an easily prepared and recyclable oxidant for synthesis of symmetrical disulfides from thiols or alkyl halides (tosylates)/thiourea. J. Sulfur Chem..

[7] Christoforou A, Nicolaou G, Elemes Y (2006). N-Phenyltriazolinedione as an efficient, selective, and reusable reagent for the oxidation of thiols to disulfides. Tetrahedron Lett..

[8] Shirini F, Zolfigol MA, Abri AR (2008). Fe (NO_3_) _3_· 9H_2_O/Fe (HSO_4_)_3_: An efficient reagent system for the oxidation of alcohols, thiols and sulfides in the absence of solvent. Chin. Chem. Lett..

[9] Vandavasi JK, Hu WP, Chen CY, Wang JJ (2011). Efficient synthesis of unsymmetrical disulfides. Tetrahedron.

[10] Yi SL, Li MC, Hu XQ, Mo WM, Shen ZL (2016). An efficient and convenient method for the preparation of disulfides from thiols using oxygen as oxidant catalyzed by tert-butyl nitrite. Chin. Chem. Lett..

[11] Banfield SC, Omori AT, Leisch H, Hudlicky T (2007). Unexpected reactivity of the burgess reagent with thiols: synthesis of symmetrical disulfides. J. Org. Chem..

[12] Nair V, Augustine A (2003). Novel synthesis of 2-arylbenzothiazoles mediated by ceric ammonium nitrate (CAN): a rebuttal. Org. Lett..

[13] Joshi G, Bhadra S, Ghosh S, Agrawal MK, Ganguly B, Adimurthy S, Ghosh PK, Ranu BC (2010). Making full use of the oxidizing equivalents in bromate in the selective oxidation of thiols, sulfides, and benzylic/secondary alcohols into disulfides, sulfoxides, and aldehydes/ketones. Ind. Eng. Chem. Res..

[14] Iranpoor N, Firouzabadi H, Pourali AR (2002). Dinitrogen tetroxide supported on polyvinylpyrrolidone (PVP–N_2_O_4_): A new nitrosating and coupling agent for thiols and a selective oxidant for sulfides and disulfides. Tetrahedron.

[15] Liu Y, Zhang Y (2003). Temperature-controlled selective reduction of arenesulfonyl chlorides promoted by samarium metal in DMF. Tetrahedron Lett..

[16] Barba F, Ranz F, Batanero B (2009). Electrochemical transformation of diazonium salts into diaryl disulfides. Tetrahedron Lett..

[17] Sun J, Xia EY, Wu Q, Yan CG (2010). Synthesis of ammonium S-S bond linked dipyridinedionates via four-component reactions of cyanoacetamide, aldehyde, amine and 1, 3-thiazolidinedione. Tetrahedron.

[18] Khalili D (2015). Graphene oxide-assisted one-pot and odorless synthesis of symmetrical disulfides using primary and secondary alkyl halides (Tosylates) and thiourea as sulfur source reagent. Phosphorus Sulfur Silicon Relat. Elem..

[19] Yu JT, Guo H, Yi Y, Fei H, Jiang Y (2014). The chan–lam reaction of chalcogen elements leading to aryl chalcogenides. Adv. Synth. Catal..

[20] Soleiman-Beigi M, Mohammadi F (2012). A novel copper-catalyzed, one-pot synthesis of symmetric organic disulfides from alkyl and aryl halides: Potassium 5-methyl-1, 3, 4-oxadiazole-2-thiolate as a novel sulfur transfer reagent. Tetrahedron Lett..

[21] Soleiman-Beigi M, Mohammadi F (2015). A novel nickel-catalyzed domino method for the direct synthesis of symmetrical disulfides using potassium 5-methyl-1, 3, 4-oxadiazole-2-thiolate as a sulfurating reagent. Synlett.

[22] Soleiman-Beigi M, Mohammadi F (2017). Simple and green method for synthesis of symmetrical dialkyl disulfides and trisulfides from alkyl halides in water; PMOxT as a sulfur donor. J. Sulfur Chem..

[23] Cao J, Li X, Tian H (2020). Metal-organic framework (MOF)-based drug delivery. Curr. Med. Chem..

[24] Jiang W, Cao JP, Xie JX, Zhao L, Zhang C, Zhu C, Zhao XY, Zhao YP, Zhang JL (2022). MOF-derived Ru@ ZIF-8 catalyst with the extremely low metal Ru loading for selective hydrogenolysis of C-O bonds in lignin model compounds under mild conditions. Catal. Sci. Technol..

[25] Veisi H, Karmakar B, Tamoradi T, Hemmati S, Hekmati M, Hamelian M (2021). Biosynthesis of CuO nanoparticles using aqueous extract of herbal tea (Stachys Lavandulifolia) flowers and evaluation of its catalytic activity. Sci. Rep..

[26] Bekru, A.G., Zelekew, O.A., Andoshe, D.M., Sabir, F.K., Eswaramoorthy, R. Microwave-assisted synthesis of CuO nanoparticles using cordia africana Lam. leaf extract for 4-nitrophenol reduction. J. Nanotechnol. **30** (2021).

[27] Ruano JL, Parra A, Alemán J (2008). Efficient synthesis of disulfides by air oxidation of thiols under sonication. Green Chem..

[28] Soleiman-Beigi M, Taherinia Z (2014). Simple and efficient oxidative transformation of thiols to disulfides using Cu (NO_3_) _2_· 3H_2_O in H_2_O/AcOEt. Monatshefte für Chemie-Chem. Mon..

[29] Alam A, Takaguchi Y, Tsuboi S (2005). Simple, extremely fast, and high-yielding oxidation of thiols to disulfides. Synth. Commun..

[30] Rosseinsky MJ (2004). Recent developments in metal-organic framework chemistry: design, discovery, permanent porosity and flexibility: metal-organic open frameworks. Microporous Mesoporous Mater..

[31] Kabalka GW, Reddy MS, Yao ML (2009). Synthesis of diaryl disulfides via the reductive coupling of arylsulfonyl chlorides. Tetrahedron Lett..

[32] Firouzabadi H, Iranpoor N, Abbasi M (2010). A one-pot, efficient, and odorless synthesis of symmetrical disulfides using organic halides and thiourea in the presence of manganese dioxide and wet polyethylene glycol (PEG-200). Tetrahedron Lett..

[33] Wang JX, Gao L, Huang D (2002). A rapid and efficient synthesis of symmetrical disulfides under microwave irradiation conditions. Synth. Commun..

[34] Hosseinzadeh R, Tajbakhsh M, Khaledi H, Ghodrati K (2007). Ethylenebis (N-methylimidazolium) chlorochromate (EBMICC): An efficient and selective reagent for the oxidation of thiols to disulfides. Monatshefte für Chemie-Chem. Mon..

