# Supplementary material for: Dipotassium 1,3,4-thiadiazole-2,5-bis(thiolate) as a new S-donor for direct synthesis of symmetrical disulfides
Source: Sci Rep. 2022 Sep 27;12:16149. doi: 10.1038/s41598-022-20642-5 (PMC9515069; doi:10.1038/s41598-022-20642-5)
Supplement: Supplementary file 1 — Supplementary Information. [file 41598_2022_20642_MOESM1_ESM.docx]

Supporting Information

**Dipotassium 1,3,4-thiadiazole-2,5-bis(thiolate) as a new S-donor for direct synthesis of symmetrical disulfides**

Mohammad Soleiman-Beigi*^a^, Mohammad Alikarami ^b^, Homa Kohzadi ^a^, Zahra Akbari ^a^

*^a^Department of Chemistry, Faculty of Science, Ilam University, P.O. Box, 69315516, Ilam, Iran.*

*^b^Department of Chemistry, Ilam Branch, Islamic Azad University, Ilam, Iran*

*Corresponding author:* *Mohammad Soleiman-Beigi*

*Tell /Fax number: +98 (843) 2227022*

*E-mail:* [*SoleimanBeigi@yahoo.com*](mailto:SoleimanBeigi@yahoo.com)


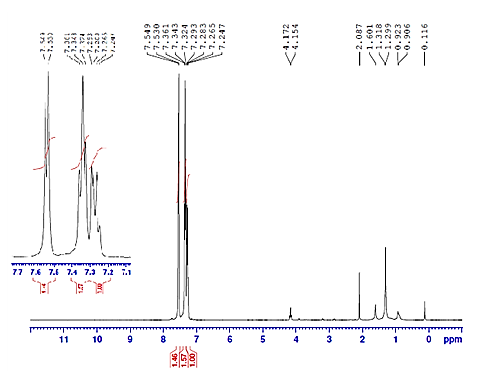


**Fig 1.** ^1^H NMR spectrum of product **2a**


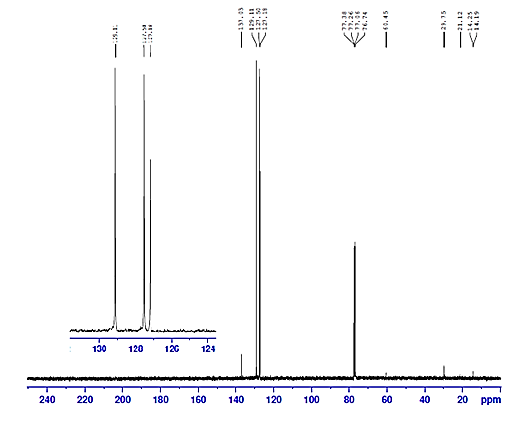


**Fig 2.** ^13^C NMR spectrum of product **2a**


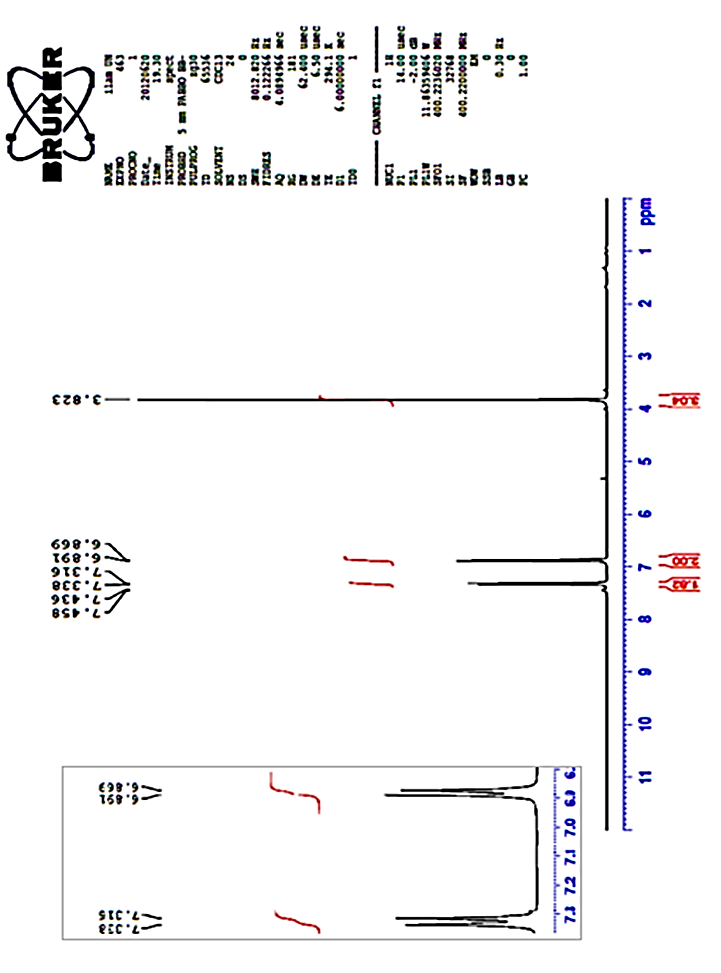


**Fig 3.** ^1^H NMR spectrum of product **2c**


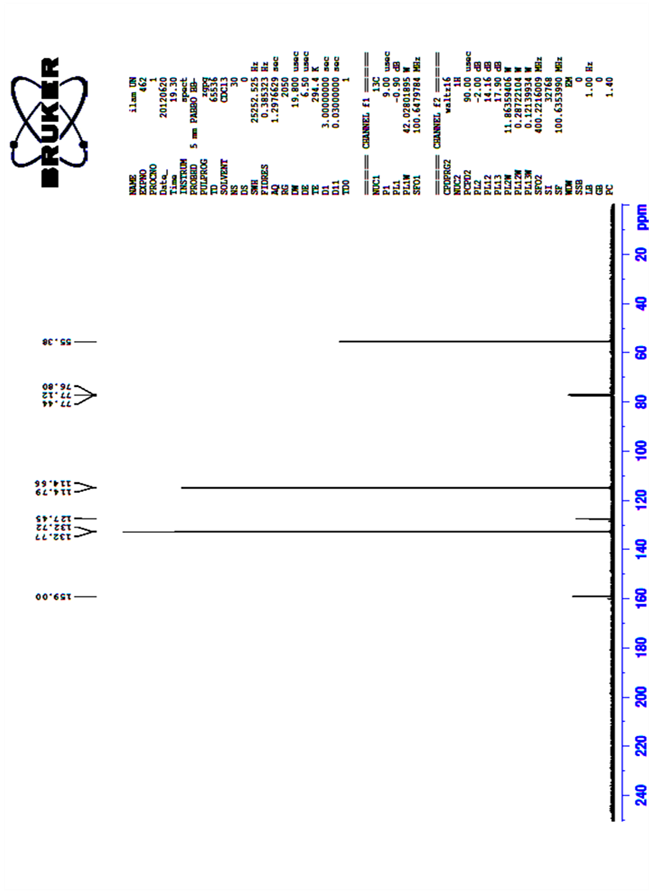


**Fig 4.** ^13^C NMR spectrum of product **2c**


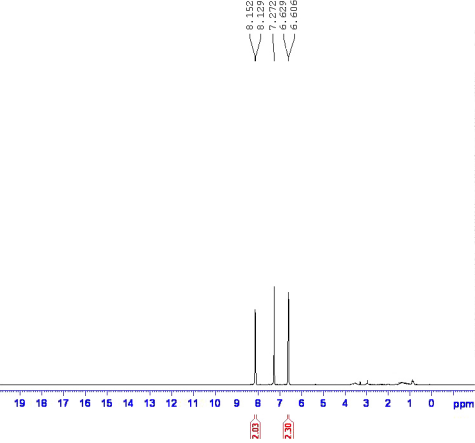


**Fig 5.** ^1^H NMR spectrum of product **2e**


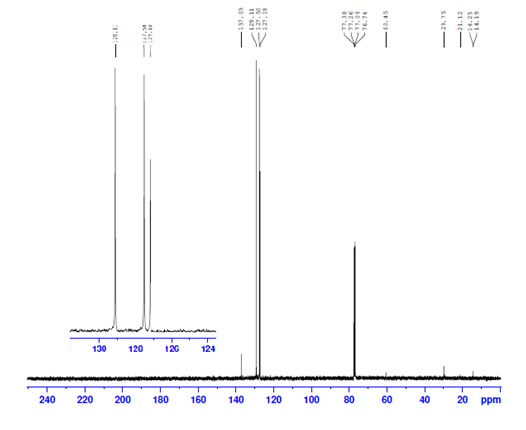


**Fig 6.** ^13^C NMR spectrum of product **2e**


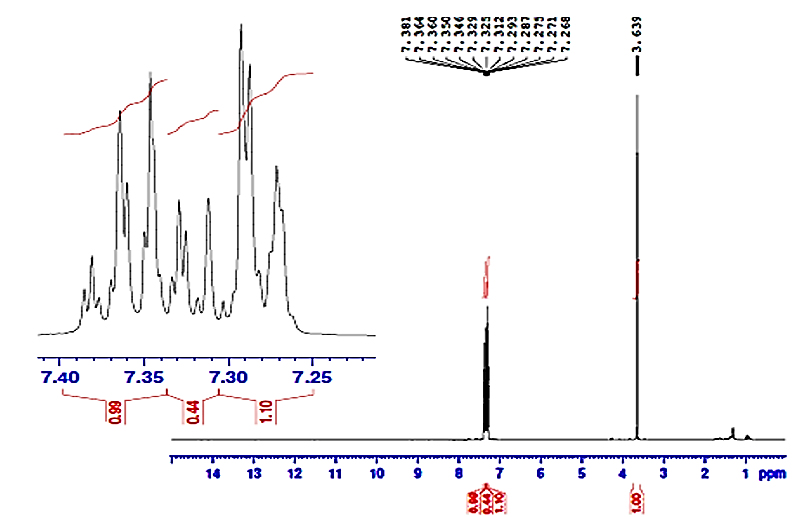


**Fig 7.** ^1^H NMR spectrum of product **2h**


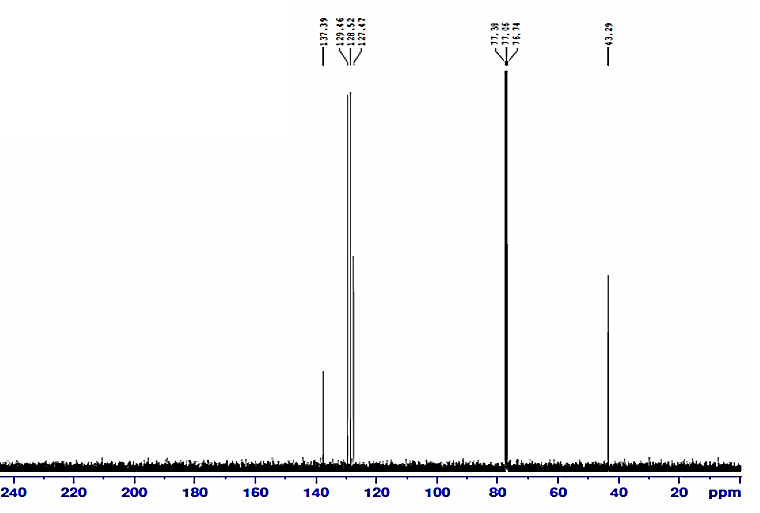


**Fig 8.** ^13^C NMR spectrum of product **2h**
